# The Fitting and Furnishing of Wards and Operating Theatres

**Published:** 1906-10-06

**Authors:** 


### THE FITTING AND FURNISHING OF WARDS AND OPERATING THEATRES.

### VENTILATION.

The ventilation of wards and of operating theatres present rather distinct features, and require in many cases separate treatment. In the case of wards, all that is required is the continual change of the air, with provision that in winter draughts are not created, and that the ventilating current does not lower the temperature in any particular part of the ward sufficiently to be uncomfortable to patients or nurses. The temperature also in the wards is required to be medium. In the operating theatre the conditions are different. The room itself is smaller, there are considerably more human beings in it per cubic foot than in the ward, and in addition to the carbonic acid expired by each human being, there are the vapours from the anæsthetic, and from the lotions, the steriliser, etc. In addition it is necessary that the temperature of the operating theatre should be rather above the medium. The conditions of the operation vary very considerably, and it is necessary that the patient shall not be exposed to a cold, or even a moderately cold atmosphere. For these reasons the artificial ventilation of operating theatres has been directed rather to the furnishing of a comparatively high temperature, and the writer understands it is sometimes found, after surgeons have been working for some hours, that the atmosphere of the room is inconveniently warm. All this, however, can easily be arranged. For the operating theatre, so far, it appears to be decided by practical experience that some form of mechanically-created ventilation is the best, principally because the air can be cleaned on its way to the operating theatre; and for the process of cleaning it must pass through some apparatus, similar to those employed in all mechanical ventilating systems. Further, where the mechanical ventilation is only directed to the operating theatre, it is possible to control the temperature and the velocity of the air from the theatre itself, by suitable arrangements, and there should be no difficulty whatever in maintaining an air current at a certain definite velocity, and yet of raising or lowering the temperature at will. This is particularly the case where the hospital has a cold storage apparatus. It is a simple matter to provide a cold brine grid, over which a portion of the air could be carried, after it has been cleaned, and from which definite quantities can be taken to mix with the main air current passing to the theatre, a sufficient time before the air enters the theatre—for the mixing to be complete—with such an arrangement a very small quantity of cold air would lower the temperature a degree or so very quickly when desired. On the other hand, there is no difficulty whatever in raising the temperature of the air current to any figure that may be desired, by passing the air over steam pipes.

For the ventilation of wards, however, as was mentioned in an article on the subject, which appeared in the columns of The Hospital some weeks ago, the mechanical ventilating systems are not so well adapted, mainly because the supply of oxygen may be less in any particular ward, or in any portion of any particular ward, than that ward or portion of it demands; while it is perfectly easy to carry warmed air into the ward, by means of radiators in front of gratings, the air being directed upwards behind the radiator, or by means of one of the stoves that have been described in the columns of The Hospital, in which the air is warmed by passing over the back of the stove on its way into the ward. Ventilation by windows and chimneys, under certain conditions of the atmosphere, is probably as efficient as can be obtained, providing that certain points are remembered. Windows on what sailors call the weather side of the ward, should not be opened except in very warm, still weather. The custom which rules in some hospitals of opening the windows on both sides of the ward is hardly to be commended. In the writer's opinion it tends to create a draught. The passage of air across either an orifice, such as the mouth of a pipe, or over another body of air, tends to draw the air up through the pipe, or from the body of air, as say, from the body of the ward, and to carry it in the direction in which the air itself is moving. Thus, when the windows opposite each other are open there will be a distinct upward draught, passing from all parts of the ward out through the windows on the lee side, that is the side away from the wind, and the draughts so created may be very unpleasant to some of the beds. Where the windows on the lee side only are opened, even when the wind is comparatively high, there will be in the great majority of cases, a gentle air current passing from the windows into the ward, finding its way down through the mass of air in the upper part of the ward, dissolving the carbonic acid and other exhalations that are rising from the beds, and carrying them harmlessly up the chimney. The cross-draught in front of the sanitary block that is often arranged appears to the writer to be very good, providing that patients are not exposed to too strong a draught in passing into and out of the ward. The frictional action of the air mentioned above will be in full force here, the air will pass in at the window on the weather side, across the front of the door of the sanitary block, and will extract the air from it, carrying it out with it, together with all the noxious germs, through the window on the lee side, the cross-air current being practically an insulator so to speak, between the sanitary block and the ward. When windows are used for ventilation the arrangement in so many hospitals of sections of the windows louvre fashion, appears to the writer to be a very good one, since the opened section of the window having its sash inclined at an angle, will direct the air upwards. Ventilation is carried on by two distinct operations, by convection currents, and by the mixing which is always going on between any two bodies of gases that are in contact. Air whose temperature is raised naturally tends to rise. On the contrary, air coming in from outside is nearly always colder than the air inside, and it tends to fall, and in doing so mixes with the air which is rising and which is charged with carbonic acid, the two being carried out by the chimney where that is the outlet. This is the reason that air entering above the heads of the inmates of a room usually gives better ventilation, providing that other arrangements are satisfactory, than the simple upward flow with cold air entering below does.

